# InSiGHT leads in the implementation of the Human Variome Project

**DOI:** 10.1186/1897-4287-9-S1-P23

**Published:** 2011-03-10

**Authors:** Finlay Macrae, Gabriela Moslein, Richard Cotton, Rolf Sijmons, Johan den Dunnen, Michael Woods, Sean Tavtigian, Mark Jenkins, Robert Hofstra, Robert Haile

**Affiliations:** 1 International Society for Gastrointestinal Hereditary Tumours (InSiGHT), http://www.insight-group.org; 2 The Human Variome Project, http://www.humanvariomeproject.org; 3 Colon Cancer Family Register, http://epi.grants.cancer.gov/CFR/; 4 Centre for Molecular, Environmental, Genetic and Analytic Epidemiology, University of Melbourne, Parkville, Victoria, Australia; 5 HELIOS, St. Josefs-Hospital, Bochum, Germany; 6 Genomic Disorders Research Centre, University of Melbourne, Parkville, Victoria, Australia; 7 Department of Genetics, University Medical Center Groningen, The Netherlands; 8 Department of Human Genetics, Leiden University Medical Center, The Netherlands; 9 Disciple of Genetics, Memorial University of Newfoundland, St. John’s, Newfoundland, Canada; 10 Department of Oncological Sciences, Huntsman Cancer Institute, Salt Lake City, Utah, USA; 11 Centre for Molecular, Environmental, Genetic and Analytic Epidemiology, University of Melbourne, Parkville, Victoria, Australia; 12 Department of Preventive Medicine, University of Southern California, Los Angeles, California, USA

## Background

With next gen sequencing, the human genome can be sequenced in a few weeks and for a few thousand dollars. Capability is no longer the issue: interpretation is. Assembling information to assist in interpreting the consequences of all base pair changes is the mission of the Human Variome Project (HVP). InSiGHT has collaborated with the HVP, and now, due its coherence and international organization, maintains a Locus Specific Database which is seen as an exemplar for the unfolding of the HVP across the whole genome.

## Aim

To develop a single comprehensive LSDB relating to MMR and other gene variants predisposing to hereditary GI cancer, permitting the most expert interpretation of the consequence of each variant through a systematic interpretation process.

## Methods

InSiGHT has appointed a range of committees to oversee a range of activities related to its LSDBs for Hereditary GI cancer. A strong collaboration has emerged between the NCI funded Colon Cancer Family Register, and InSIGHT, extending even further by invitation to all parties interested in MMR repair – the INSILICO MMR consortium. The work is bonded together through the development of the Bayesian Likelihood Ratio for interpretation of VUS, in a transatlantic and Australasian effort.

## Results

In the last two years, submissions have increased from 550 to over 13000. The InSIGHT MMR database attracts over 20,000 hits per month from interested parties. All the major MMR databases have been incorporated onto the InSiGHT MMR database (InSiGHT, Newfoundland, functional assay databases) and there have been large depositions of national data (German, French, Canadian, Chinese). A phenotype dataset is being finalized, assisted by the NCI. Functional studies are being assimilated and further developed especially from European expertise. The Bayesian Likelihood Ratio approach to assigning pathogenicity informed by the range of information available relevant to this, is being lead by Sean Tavtigian, sponsored by IARC and now in Utah. The interpretation committees are being supported by the Cancer Council of Victoria. Recently, funding has been secured from the Melbourne based Hicks Foundation for a full time curator of the MMR database. The largest assembly of MMR families ever is associated with the INSILICO consortium, underpinning a range of research opportunities.

## Conclusion

InSiGHT is proud of its achievements and leadership for the HVP. Members of the CGA are encouraged to support the InSiGHT databases, so the world can share information on variants for the general good and support of families with MMR mutations.

